# Three-Dimensional Self-assembled Hairball-Like VS_4_ as High-Capacity Anodes for Sodium-Ion Batteries

**DOI:** 10.1007/s40820-020-0377-7

**Published:** 2020-01-25

**Authors:** Shuangshuang Ding, Bingxin Zhou, Changmiao Chen, Zhao Huang, Pengchao Li, Shuangyin Wang, Guozhong Cao, Ming Zhang

**Affiliations:** 1 Key Laboratory for Micro/Nano Optoelectronic Devices of Ministry of Education, Hunan Provincial Key Laboratory of Low-Dimensional Structural Physics and Devices, School of Physics and Electronics, Hunan University, Changsha, 410082 People’s Republic of China; 2 College of Electrical Engineering and New Energy, Three Gorges University, Yichang, 443002 Hubei People’s Republic of China; 3 State Key Laboratory of Chemo/Biosensing and Chemometrics, Provincial Hunan Key Laboratory for Graphene Materials and Devices, College of Chemistry and Chemical Engineering, Hunan University, Changsha, 410082 People’s Republic of China; 4 Department of Materials Science and Engineering, University of Washington, Seattle, WA 98195 USA

**Keywords:** VS_4_, Sodium-ion batteries, Low-temperature batteries, Reaction kinetics, Na^+^ storage mechanism

## Abstract

Sodium-ion batteries (SIBs) are considered to be attractive candidates for large-scale energy storage systems because of their rich earth abundance and consistent performance. However, there are still challenges in developing desirable anode materials that can accommodate rapid and stable insertion/extraction of Na^+^ and can exhibit excellent electrochemical performance. Herein, the self-assembled hairball-like VS_4_ as anodes of SIBs exhibits high discharge capacity (660 and 589 mAh g^−1^ at 1 and 3 A g^−1^, respectively) and excellent rate property (about 100% retention at 10 and 20 A g^−1^ after 1000 cycles) at room temperature. Moreover, the VS_4_ can also exhibit 591 mAh g^−1^ at 1 A g^−1^ after 600 cycles at 0 °C. An unlike traditional mechanism of VS_4_ for Na^+^ storage was proposed according to the dates of ex situ characterization, cyclic voltammetry, and electrochemical kinetic analysis. The capacities of the final stabilization stage are provided by the reactions of reversible transformation between Na_2_S and S, which were considered the reaction mechanisms of Na–S batteries. This work can provide a basis for the synthesis and application of sulfur-rich compounds in fields of batteries, semiconductor devices, and catalysts.

**Electronic supplementary material:**

The online version of this article (10.1007/s40820-020-0377-7) contains supplementary material, which is available to authorized users.

## Highlights

- The unique hairball-like VS_4_ composed of spiral nanowires was successively constructed through a one-step hydrothermal method.
- The prepared hairball-like VS_4_ exhibits high capacity and long cycle stability for Na^+^ storage at room temperature, and it can tolerate drastic temperature changes.
- The ex situ characterization and electrochemical kinetic analysis reveal that the storage mechanisms of VS_4_ changed with the increase in the number of cycles.

**Electronic supplementary material:**

The online version of this article (10.1007/s40820-020-0377-7) contains supplementary material, which is available to authorized users.

## Graphical abstract

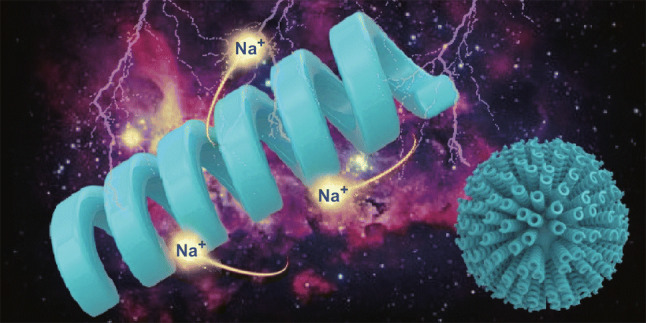

## Introduction

Since the commercialization of lithium-ion batteries (LIBs) in 1991, its lightweight, high energy density, and long life cycle have performed significant progress in the applicability of portable electronic products using LIBs as energy storage devices [1–3]. However, due to the limited and uneven distribution characteristics of lithium resources, the cost of this device category has significantly increased [4–6]. Therefore, it is essential to look for alternative approaches. Recently, sodium-ion batteries (SIBs) have received extensive attention as a promising potential alternative to LIBs owing to the inherent richness and widespread distribution of sodium resources [7–9]. SIBs and LIBs have similar working principles. However, due to the relatively large ionic radius (1.02 Å for Na^+^ vs. 0.76 Å for Li^+^) and the mass of Na^+^ ions, weak rate capability and the drastic capacity decay can occur. Hence, developing active materials, capable of adapting to the rapid and stable insertion/extraction of Na^+^, can be the key to promoting the development of SIBs [10–12].

In order to develop appropriate anode materials of the SIBs, several compounds (including hard carbon [13–15], metals and alloys [16–18], metal oxides [19, 20], and metal chalcogenides [21–24]) have been widely studied. In particular, transition metal sulfides (TMSs) can be considered to be promising materials, mainly due to their unique physicochemical properties (high conductivity, consistent thermal stability, high soil abundance, etc.), higher capacity compared to carbon-based materials, and safer working potential. Moreover, they can provide favorable real-life conditions for batteries with low development cost and excellent performance [25–27]. It is worth noting that VS_2_ with a sandwich structure possesses a layer spacing of 5.76 Å, which can enable efficient insertion/de-intercalation of Na^+^, and high theoretical capacity and exhibits stable electrochemical performance [28, 29]. Compared with VS_2_, VS_4_ is another material with the same state of vanadium (V^4+^), which has attracted considerable attention due to its higher theoretical capacity (1196 mAh g^−1^) [30, 31]. The higher capacity is owing to the higher content of the S element that is the electrochemically active element and provides abundant active sites [32, 33]. Historically, VS_4_ was first discovered in green minerals in 1906, and its crystal structure was elucidated in 1964 [34, 35]. It is known that VS_4_ has a one-dimensional chain crystal structure composed of S_2_^2−^ dimers and the inter-chain distance is 5.83 Å, which not only provides more active sites for alkaline ions but also can promote more efficient charge transfer [36, 37]. For example, the flower-like VS_4_/rGO composites can deliver a reversible capacity of 80 mAh g^−1^ at a current density of 0.1 A g^−1^ after 100 cycles when used as cathodes for aluminum-ion batteries [38]. When used as the anode for LIBs, the VS_4_ nanoparticles rooted by carbon nanotubes provide a reversible capacity of 922 mAh g^−1^ with a current density of 0.5 A g^−1^ and also can exhibit remarkable chemical properties in SIBs [39]. Although VS_4_ has received extensive attention, pure VS_4_ has been rarely studied as active materials for the secondary batteries, and corresponding mechanism for Na^+^ storage also was not clear.

Herein, pure hairball-like VS_4_ composed of spiral nanowires was successfully prepared through a simple template-free hydrothermal method and its morphological evolution mechanism is investigated by controlling the reaction time. The optimized hairball-like VS_4_ as anodes materials for SIBs showed an outstanding electrochemical property (660 and 589 mAh g^−1^ at 1 and 3 A g^−1^, respectively) at room temperature. Furthermore, the VS_4_ electrodes exhibited excellent discharge capacity (591 mAh g^−1^ at 1 A g^−1^) and long-term cyclability (320 mAh g^−1^ at 3 A g^−1^ after 600 cycles) at 0 °C. Ex situ characterization, cyclic voltammetry method, and electrochemical kinetic investigations were utilized to elucidate the mechanism of Na^+^ storage. In this paper, research on the hairball-like VS_4_ as anodes for SIBs can provide essential ideas for the future selection of rechargeable batteries active materials.

## Experimental Section

### Synthesis of VS_4_

All chemicals used were of analytical grade and were used as received without any further purification and modification. In a typical procedure, 0.36 g vanadium source (Na_3_VO_4_) and 2.0 g sulfur source (CH_3_CSNH_2_) were first dissolved in deionized water, followed by the stirring of the solution for 1 h before applying the hydrothermal treatment in a 50-mL stainless steel autoclave, sealed, and kept at 160 °C for 24 h. The final obtained products were collected by centrifugation, washed in deionized water and ethanol several times, and then dried at 70 °C for 12 h in an oven.

### Material Characterization

The samples were characterized by scanning electron microscopy (SEM, S4800 at 5 kV) and transmission electron microscopy (TEM, JSM-2100 at 200 kV), X-ray diffraction (XRD, Siemens D5000, Cu Kα, *λ* = 0.15406 nm), thermogravimetric analysis (TGA, Shimadzu DTG-60), Raman spectra (laser wavelength of 512 nm), and X-ray photoelectron spectroscopy (XPS, VG MultiLab 2000).

### Electrochemical Measurements

The electrodes were obtained by mixing active materials (70 wt%), conductivity agent carbon black (20 wt%), and polyvinyl difluoride (10 wt%) together and ground with an agate mortar, and then, the right amount of *N*-methylpyrrolidone was added to form a slurry. Then, the slurry was coated onto a copper foil and dried in the vacuum box at 70 °C for 12 h. The copper foil was cut into disks with a diameter of 12 mm, and the loading density of active material was 0.9–1.1 mg cm^−2^. The CR2025-type coin cells were assembled in an argon-filled glove box (M. Braun, Germany) with H_2_O and O_2_ levels less than 0.5 ppm, using Na foils as counter electrode and glass fibers as separators. The electrolyte was 1 M sodium trifluoromethanesulfonate (NaSO_3_CF_3_) in the diglyme (DGM). Besides, the low-temperature (0 °C) tests were performed under a refrigerator. The galvanostatic charging/discharging cycling stability and rate performance tests of the batteries were performed at the cut-off voltage of 0.2–3.0 V (vs. Na^+^/Na) by using an Arbin battery cycler (BT2000). All the energy densities were calculated based on the mass of anode active material. The cyclic voltammetry (CV) and electrochemical impedance spectroscopy (EIS) were tested on a CHI 660E (Chenhua) electrochemical workstation system.

## Results and Discussion

**Fig. 1:**
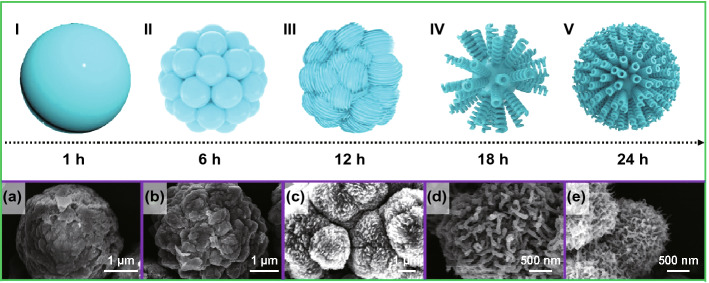
Schematic illustrations of the time-dependent hydrothermal reaction: self-sacrificed evolution mechanism from microsphere to hairball-like structure

Figure 1 shows the detailed schematic of time-dependent experiments to investigate the morphological transformation mechanism of the VS_4_ from spheres to hairball-like structures. When the hydrothermal treatment time was 1 h, the original microspheres with a diameter of 3–4 μm were formed due to the rapid nucleation and crystal growth process, as shown in Fig. 1a. As the reaction time of hydrothermal treatment extended to 6 h, the surface of the microspheres fissured many small balls due to the Ostwald ripening (Fig. 1b**)** [40]. With the further increase in treatment time (to 12 h), the metastable balls on the surface were used as a self-success template and were dissolved and recrystallized into nanofibers owing to the proximity to the reaction medium (Fig. 1c). This behavior can be attributed to the fact that as the reaction proceeds, the thioacetamide is further decomposed, increasing the production of NH_3_, and the Ostwald ripening effect is more accentuated. As the treatment time increased to 18 h, more nanofibers appeared, and they became longer and more curled, and they agglomerated together to form a ball about 2 μm (Fig. 1d). Finally, as the hydrothermal time increases to 24 h, the nanofibers become more curved and smaller in size, thereby agglomerating into smaller hairball-like microspheres, as shown in Fig. 1e. In addition, the morphology of the material cannot be significantly optimized when the reaction time is continuously increased, as shown in Fig. S1.

**Fig. 2:**
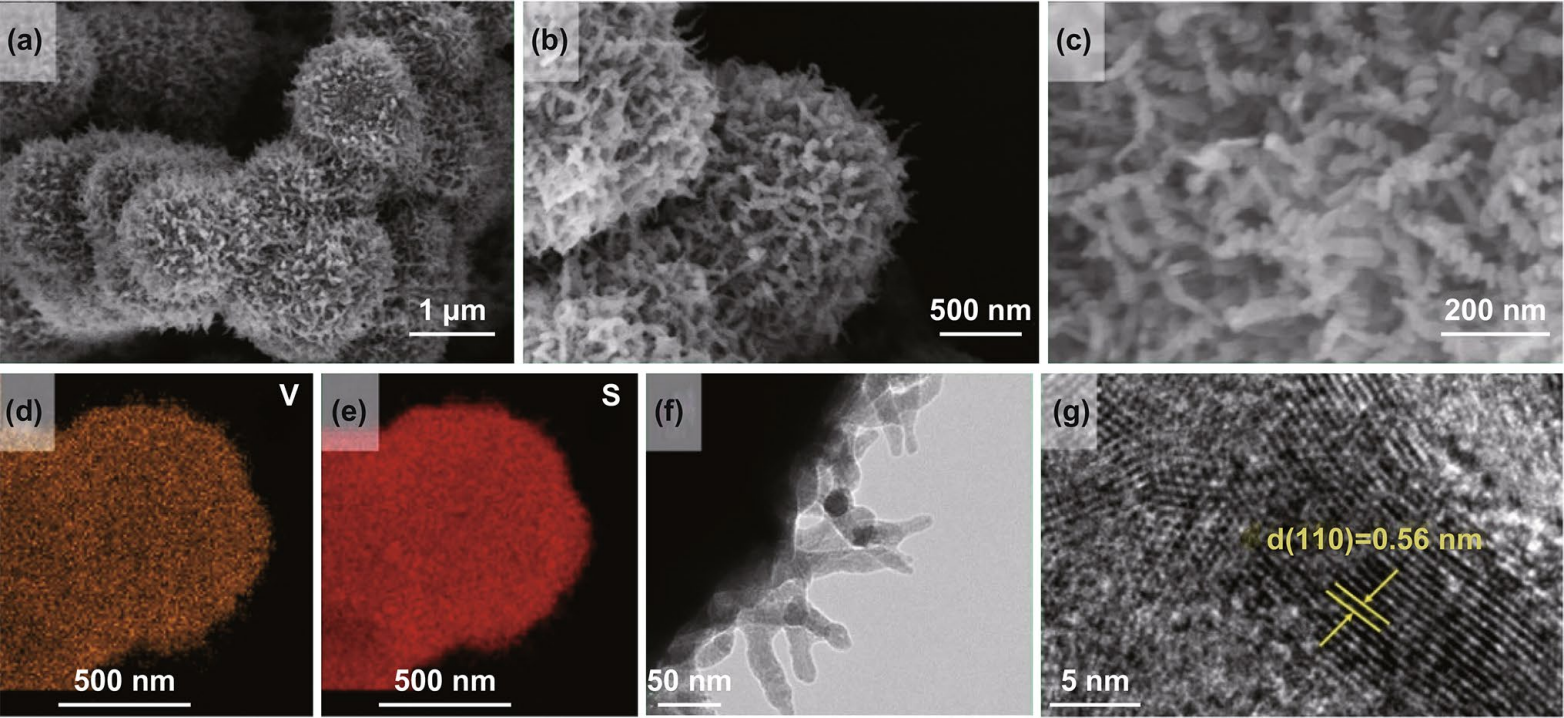
The morphology characterizations of hairball-like VS_4_. **a**–**c** SEM images, **d**, **e** TEM element mappings analysis of VS_4_, clearly indicating that the V, S elements are uniform distribution along the VS_4_ nanosphere. **f**, **g** TEM and HRTEM images of VS_4_

The morphology of VS_4_ was characterized by SEM and TEM. The lateral view (Fig. S2a) and vertical view (Fig. S2b) of the VS_4_ indicated it is linear chain. Previous investigations have shown that the forces between the atomic chains of VS_4_ are the relatively weak van der Waals forces, the spacing between atomic chains being 5.83 Å, which is much larger than the size of Na^+^ (1.02 Å), showing a narrow band gap of 1.0 eV with relative higher electronic conductivity [41]. Figure 2a–c reveals the morphology of VS_4_ with a uniform size of about 1 μm assembled by spiral nanowires with an average diameter of about 150 nm. The TEM element mapping analysis confirmed the uniform distribution of different elements (including V and S) in the hairball-like VS_4_ nanostructures (Fig. 2d, e). Moreover, the TEM image (Fig. 2f) shows the thorn above the VS_4_ nanosphere with a diameter of about 20 nm. Figure 2g shows the high-resolution TEM (HRTEM) image taken from a nanofiber and the spacing between adjacent lattice faces along a specific direction is 0.56 nm, corresponding to the (110) plane of the hairball-like VS_4_ nanostructures. In addition, the selected area electron diffraction (SAED) pattern (Fig. S3) confirms that the hairball-like VS_4_ structures have a polycrystalline nature. According to the adsorbent–desorption curves in Fig. S4, the surface area of the hairball-like VS_4_ is calculated to be 62 m^2^ g^−1^.

**Fig. 3:**
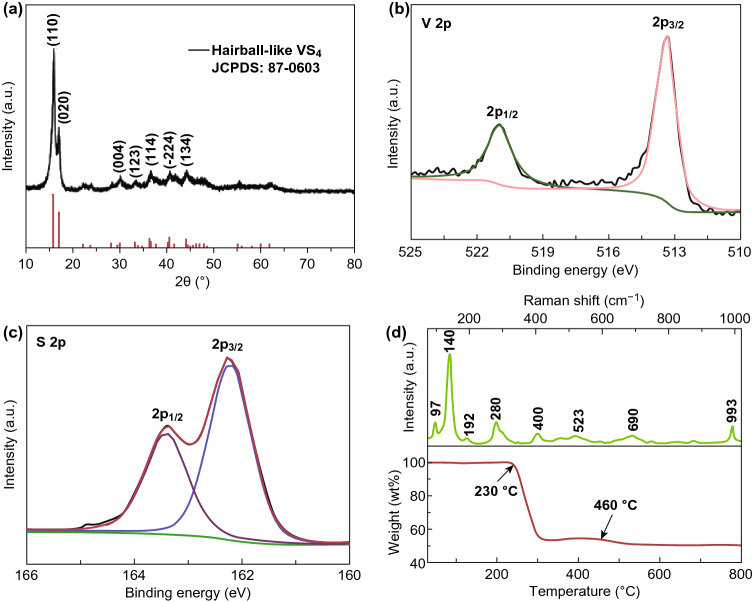
The composition characterizations of hairball-like VS_4_. **a** XRD pattern, XPS spectra of **b** V 2*p* and **c** S 2*p*, **d** Raman spectrum and TGA spectrum

The crystal structure of the hairball-like VS_4_ was examined by XRD, as shown in Fig. 3a. All characteristic diffraction peaks are in good agreement with the pure phase of VS_4_ (JCPDS card no. 87-0603, space group I2/*c*, *a* = 6.78 Å, *b* = 10.42 Å, *c* = 12.11 Å) without notable impurity [42, 43]. The main diffraction peaks at 15.8° and 17.0° correspond to the (110) and (020) diffraction planes, and their interplanar spacing (calculated according to the Bragg equation) is 0.56 and 0.52 nm. To understand deeper and to quantify the chemical composition of the VS_4_, the samples were investigated by the XPS, TGA, and Raman analysis, and the results are shown in Fig. 3b–d. In the V 2*p* XPS spectra, the V 2*p*_3/2_ peak at 513.4 eV and the V 2*p*_1/2_ peak at 520.9 eV confirmed the presence of V^4+^ [44, 45]. The high-resolution XPS scanning at S 2*p* range can be divided into two characteristic peaks: 2*p*_1/2_ (163.4 eV), 2*p*_3/2_ (162.2 eV), which are pointing to the S_2_^2−^, as it is shown in Fig. 3c [46, 47]. As shown in Fig. 3d, these peaks are located at 97,140, 192, 280, 400, 523, 690, and 993 cm^−1^, being attributed to the stretching and bending bands of VS_4_ [48, 49]. In addition, the at.% ratio of V/S was calculated to be 1:4 according to the energy-dispersive spectrometer data, further confirming the successful preparation of VS_4_ (Fig. S5). More information about the thermal decomposition of VS_4_ in air is shown in Fig. 3d, and the decomposition was carried out in three steps. In the first step (30–230 °C), the mass reductions were attributed to the desorption of the water present in the system. In the second step in the range of 230–460 °C, a weight loss (~ 45 wt%) was observed, caused by the two-step decomposition of VS_4_. The loss of mass in the range of 230–300 °C indicated the reaction of VS_4_ with O_2_, and the loss of mass in the range of 300–460 °C was associated with the reaction between the sulfuric compounds and O_2_ [50]. At higher temperatures (460–800 °C), the mass reductions were attributed to the volatilization and decomposition of vanadium oxide. In addition, the crystal structure of the prepared VS_4_ heated to 450 °C was characterized by XRD, as shown in Fig. S6. The main diffraction peaks are in good agreement with the PDF standard card (JCPDS card no. 41-1426) of V_2_O_5_, which also proves that the second-stage mass attenuation is mainly attributed to the conversion of VS_4_–V_2_O_5_.

**Fig. 4:**
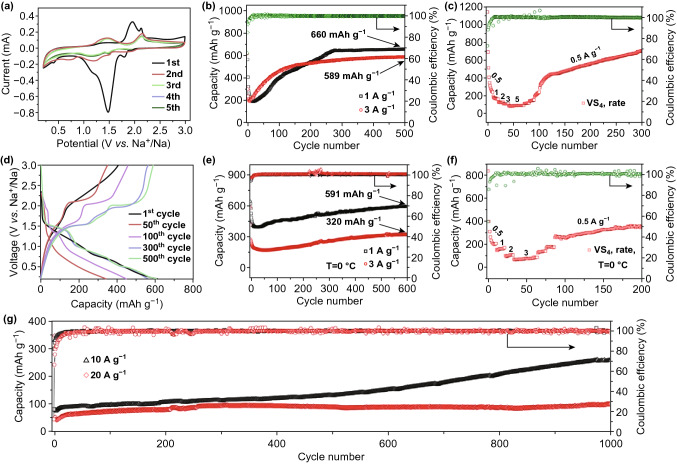
The electrochemical performances of SIBs with VS_4_ as anodes. **a** CV curves between 0.2 and 3 V at a scanning rate of 0.2 mV s^−1^. **b** Cycling properties of at different current densities of 1 and 3 A g^−1^. **c** Rate property at various current densities (from 0.5 to 5 A g^−1^) at room temperature. **d** Charge/discharge profiles at a current density of 3 A g^−1^. **e** Long-time stability at 0 °C with different current densities (1 and 3 A g^−1^). **f** Rate performance at various current densities (from 0.5 to 5 A g^−1^) at a test temperature of 0 °C. **g** Long-term cyclic stability at high current densities of 10 and 20 A g^−1^

The electrochemical redox properties of the hairball-like VS_4_ as anodes for SIBs were evaluated using CV curves. Figure 4a shows the CV curves between 0.2 and 3 V at a scanning rate of 0.2 mV s^−1^. At the initial cathodic scan, the reduction peaks of 1.45 and 1.95 V may be assigned to the sodiumation process of VS_4_ to Na_*x*_VS_4_. Moreover, the peak being observed at 0.4 V can be attributed to the decomposition of electrolyte, the formation of a solid-electrolyte interphase (SEI) film, and the reduction of Na_*x*_VS_4_ to Na_2_S and V. In the initial anodic scan, the oxidation peaks of 1.95 and 2.15 V can correspond to the de-intercalation of Na^+^ from Na_2_S and the formation of the VS_4_ or Na_*x*_VS_4_ [30, 39]. In subsequent cycles, the redox peaks shift slightly forward direction, which can be attributed to the compositional changes and structural rearrangements. From third to fifth cycles, the curves are almost coincident, indicating good cyclical stability of the discharge/charge processes.

Appropriate cut-off voltage is an important factor which affects battery performance. To optimize the electrochemical properties of the VS_4_ anode electrode, three cut-off voltages were tested to improve Na^+^ storage, as shown in Fig. S7. The cycling performance of SIBs using VS_4_ as anode material was evaluated by recording the discharge capacities between 0.2 and 3.0 V at the current densities of 1 and 3 A g^−1^, as shown in Fig. 4b. At the current densities of 1 and 3 A g^−1^, the batteries delivered high specific capacity and excellent cycle properties (660 and 589 mAh g^−1^, respectively) after 500 cycles with an initial high Coulombic efficiency (CE) of 99.9%. The discharge capacity curve gradually increases to stability after a sharp decline, which is different from the previous results about the VS_4_. The increase in capacity can be attributed to the fact that with increasing number of cycles, the contact area of VS_4_ electrode with the electrolyte became more extensive, and the number of active sites increased, thus enhancing the charge transfer during the charge/discharge processes and improving the electrochemical performance [29]. The storage mechanism of Na^+^ in VS_4_ electrode will be explained in detail later. What is more, the SEM diagrams of VS_4_ electrode after different cycles at the full-charge stages in Fig. S8 show that the original smooth surface becomes coarse rough as the number of cycles increases while there is no obvious pulverization in the structure. Figure 4c shows that the rate capability of VS_4_ at different current densities range from 0.5 to 5 A g^−1^. The discharge capacity can increase to 705 mAh g^−1^ when the current density returns to 0.5 A g^−1^, which indicating excellent reversibility. Figure 4d shows the discharge/charge platforms of VS_4_ at a current density of 3 A g^−1^, which are in good agreement with the CV curves. In addition, the performance of the battery can also be expressed by the temperature test. Figure 4e shows a high reversible discharge capacity and excellent capacity retention for Na^+^ (591 and 320 mAh g^−1^ at 1 and 3 A g^−1^ after 600 cycles) at a test temperature of 0 °C. The rate property of the SIBs was recorded the discharge capacities with the increase in current density from 0.5 to 5 A g^−1^ at a test temperature of 0 °C, as shown in Fig. 4f. It can be seen that the VS_4_ as the anodes of the batteries can withstand severe temperature changes and maintain excellent performance. This low-temperature storage behavior of Na^+^ can provide a basis for investigations of the operating temperature of the batteries. Simultaneously, it can be seen that the reversible high capacities of VS_4_ electrode are 258 and 101 mAh g^−1^ after 1000 cycles with the excellent initial Coulombic efficiency (CE) of about 100% at high current densities of 10 and 20 A g^−1^ at room temperature (Fig. 4g), respectively, suggesting significant properties of VS_4_ among the already-reported V-based sulfide (Table S1). This is due to its unique reaction mechanism in the batteries during discharging/charging processes, followed by detailed research.

**Fig. 5:**
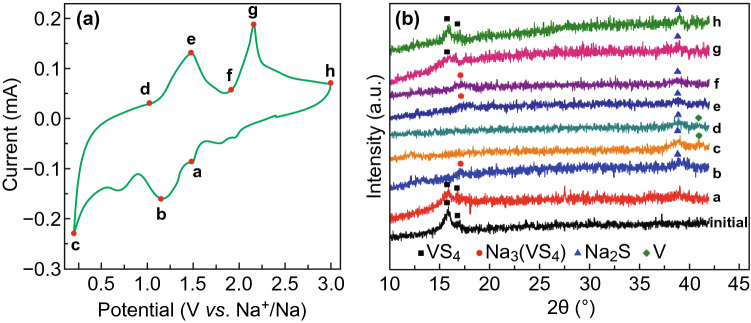
The Na^+^ storage mechanism of VS_4_ electrode in the second cycle: **a** the different discharge and charge states for ex situ XRD at second CV curve, **b** the ex situ XRD patterns of VS_4_ electrode as anodes of SIBs during the discharging and charging process

In order to further investigate the storage mechanism of Na^+^ in the VS_4_ electrode, ex situ XRD analysis was performed during the second cycle at a current density of 50 mA g^−1^. *Ex situ* XRD images of VS_4_ electrode after charged/discharged to different voltage states are shown in Fig. 5. The two peaks of the initial electrode showing at 15.8° and 17.0° are the main peaks of the pure phase of VS_4_. When discharged to 1.2 V (point b), the main peak of VS_4_ decreased, and the peaks of Na_3_VS_4_ (JCPDS No. 88-0032) appeared, suggesting that Na^+^ was inserted in the structure of VS_4_, forming the Na_x_VS_4_ (${\text{VS}}_{4} + {\text{XNa}}^{ + } + {\text{Xe}}^{ - } \to {\text{Na}}_{x} {\text{VS}}_{4}$). With the decrease in discharging potential, the intensity of the peak indexed to Na_2_S (JCPDS no. 18-1257, 38.9° attributing to the (220)) became stronger. When the discharging procedure stopped at 0.2 V (point c), the main peak of Na_3_VS_4_ disappeared, and a new peak at 41.2° corresponding to the (111) crystallographic plane of the metallic V (JCPDS No. 88-2322) appeared except the peak of Na_2_S. This implied that the VS_4_ phase has been converted to metal V and Na_2_S (${\text{Na}}_{x} {\text{VS}}_{4} + \left( {8 - X} \right) {\text{Na}}^{ + } + \left( {8 - X} \right){\text{e}}^{ - } \to 4{\text{Na}}_{2} {\text{S}} + {\text{V}}$) during the discharge process, while, during the desodiation process, Na_2_S may not be converted entirely to VS_4_, which can also be explained by the weakening of the relative intensity of the peaks of Na_3_VS_4_ (charge to 1.4 V (point e) and 1.8 V (point f)). When the charging procedure was stopped at 3.0 V (point h), the main peaks of VS_4_ appeared again, but the peak of Na_2_S did not disappear entirely, which proves again that the capacity is reduced at the beginning.

**Fig. 6:**
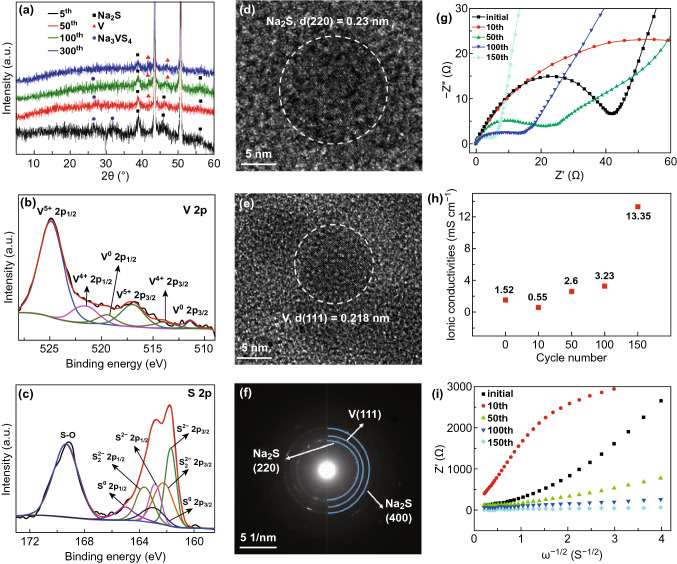
**a** The ex situ XRD patterns of VS_4_ electrodes with a current density of 3 A g^−1^ at fully charged states after 5, 50, 100, and 300 cycles. The high-resolution XPS spectra of **b** V 2*p* and **c** S 2*p* after the 100 cycles at the full-charge stage. **d**–**f** TEM and SAED images of VS_4_ electrodes after the 100 cycles at the full-charge stage. **g** Nyquist plots of VS_4_ electrodes after different cycles with a current density of 3 A g^−1^. **h** Ionic conductivity values of the batteries with different numbers of cycles at a current density of 3 A g^−1^. **i** Warburg factor of the batteries with a current density of 3 A g^−1^ after different cycles

In order to further investigate the electrochemical reaction mechanism of VS_4_ electrodes, ex situ XRD (Fig. 6a) was performed at a current density of 3 A g^−1^ at fully charged states after 5, 50, 100, and 300 cycles, respectively. When electrodes were fully charged to 3 V (after five cycles), the XRD results showed the presence of the peaks of Na_3_VS_4_ and Na_2_S, indicating that the reaction shown in Fig. 5 is partially reversible and this is attributed to the capacity decay in the initial few cycles (Fig. 4b). As the number of cycles increases (after 50 cycles), the intensities of the peaks of Na_3_VS_4_ and Na_2_S gradually became smaller, and a new peak at 41.2° appeared, attributed to the (111) crystallographic plane of the metallic V appeared. When the number of cycles is further increased to 100, the peaks’ intensities of Na_3_VS_4_ and Na_2_S are reduced further. When the number of cycles reached 300, the intensities of the peaks of Na_3_VS_4_ were almost completely disappeared, and the intensities of the peaks of metal V became even stronger. Therefore, according to the results mentioned above, a mechanism where the VS_4_ electrodes undergo a three-step separation mechanism during the discharging/charging processes was proposed. During the initial ten cycles, the transformation of VS_4_ to Na_2_S and V is partially reversible, so the discharge capacities of the batteries are attenuated. A subsequent increase in capacity can be owed to the increment of the reversible transformation reaction between Na_2_S and S to store the Na^+^, and the presence of metal vanadium which can improve the conductivity of the electrode during the cycles. The capacities of the final stabilization stage can be attributed to the reversible conversion reaction between Na_2_S and S which was the main reaction mechanism of Na–S batteries [31, 51].

XPS and TEM analysis were performed to further investigate the electrochemical reaction mechanisms of the VS_4_ electrode at the fully discharged state after 100 cycles. In the V 2*p* XPS spectrum (Fig. 6b), the V 2*p*_1/2_ peak at 524.4 eV and the V 2*p*_3/2_ peak at 516.6 eV confirmed the presence of V^5+^, which can be attributed to the partial oxidation of V^4+^ [46]. Two peaks at 519.1 and 510.9 eV are ascribed to V^0^ 2*p*_1/2_ and V^0^ 2*p*_3/2_, respectively, proving the presence of metallic V [51]. In addition, the XPS spectrum of S 2*p* (Fig. 6c) can consist of seven peaks: (1) two peaks at 163.4 and 162.2 eV are ascribed to S_2_^2−^ 2*p*_1/2_ and S_2_^2−^ 2*p*_3/2_, respectively; (2) the other two peaks at 162.8 and 161.7 eV are indexed to S^2−^ 2*p*_1/2_ and S^2−^ 2*p*_3/2_, respectively; (3) surprisingly, the peaks at 165.1 and 163.1 eV are attributed to S^0^ 2*p*_1/2_ and S^0^ 2*p*_3/2_, respectively. The above results confirmed the presence of amorphous sulfur [51]. In Fig. 6d, e, the lattice fringes can be observed with the *d*-spacing of 0.23 and 0.218 nm, consisting of the (220) and (111) planes of Na_2_S and metal V, respectively. The diffused diffraction rings of the SAED patterns (Fig. 6f) can be indexed to Na_2_S and metal V. The XPS, TEM, and SAED results are consistent with each other, suggesting the existence of the metal V after 100th cycle (even at a large scale), implying the reasonability of the reaction between Na_2_S and S to store Na^+^ after 100 cycles.

The internal resistances of VS_4_ electrodes after different cycles were further analyzed by EIS (Fig. 6g). The Nyquist plots of VS_4_ electrodes after different cycles are presenting a semicircle in the high-frequency range and a slash line in the low-frequency range. The charge transfer resistances (*R*_ct_) of batteries are 45.7, 125.9, 26.7, 21.5, and 5.2 Ω for initial, 10th, 50th, 100th, and 150th cycles, respectively. It can be found that the values of *R*_ct_ are increased at the stage of the capacity decreased, and gradually decreased at the subsequent stage.

The values of ionic conductivity (*σ*) can be calculated by Eq. 1:

1 $$\sigma = \frac{d}{AR}$$

where *d* and *R* are the thickness and resistances of materials, respectively, and *A* is the area of the electrodes. The measurements showed that the thickness of the material was about 1 mm and the area of the electrode was 1.44 cm^2^. Therefore, ionic conductivity values (Fig. 6h) of the batteries with different numbers of cycles are 1.52, 0.55, 2.6, 3.23, and 13.35 mS cm^−1^, respectively. The increase in *σ* in the 100th and subsequent cycles can be attributed to the presence of metal *V*, according to the results from Fig. 6e, f.

The sodium diffusion coefficient (*D*_Na+_) of batteries after different cycles was also calculated by using Eq. 2 [52–54]:

2 $$D_{{{\text{Na}}^{ + } }} = R^{2} T^{2} /2A^{2} n^{4} F^{4} C^{2} \sigma^{2}$$

where *R* is the gas constant, *T* is the absolute temperature, *A* is the surface area of the anode, *n* is the number of electrons transferred, *F* is the Faraday constant, *C* is the concentration of Na^+^ in the solid (calculated as the stoichiometric amount of lithium per active mass of the VS_4_ divided by the volume of battery), and *σ* is the Warburg factor, which can be described by Eq. 3:

3 $$Z^{\prime } = R_{D} + R_{L} + \sigma \omega^{{{\raise0.7ex\hbox{${ - 1}$} \!\mathord{\left/ {\vphantom {{ - 1} 2}}\right.\kern-0pt} \!\lower0.7ex\hbox{$2$}}}}$$

where Z′ is the real resistance and angular frequency *ω *= 2*πf* (f is the frequency in low-frequency range). As shown in Fig. 6i, the Warburg factor of batteries after different cycles is 459, 1169, 164, 45, and 7, respectively. Obviously, the *D*_Na+_ of VS_4_ electrodes increase in first few cycles and decrease after 10th cycles. This tendency is contrasting with that of *R*_ct_. Generally, the low *R*_ct_ and Warburg factor, and the high ionic conductivity values are beneficial to the Na^+^ storage with high capacity at a large current density. The data mentioned above show that the change tendency of specific capacity is in contrast to that of ionic conductivity and is contrasting with *R*_ct_ and Warburg factor. Therefore, the decrease in initial ten cycles and the increase in the following cycles of specific capacities in Fig. 4b can be attributed to different storage mechanisms of VS_4_ at different cycles.

**Fig. 7:**
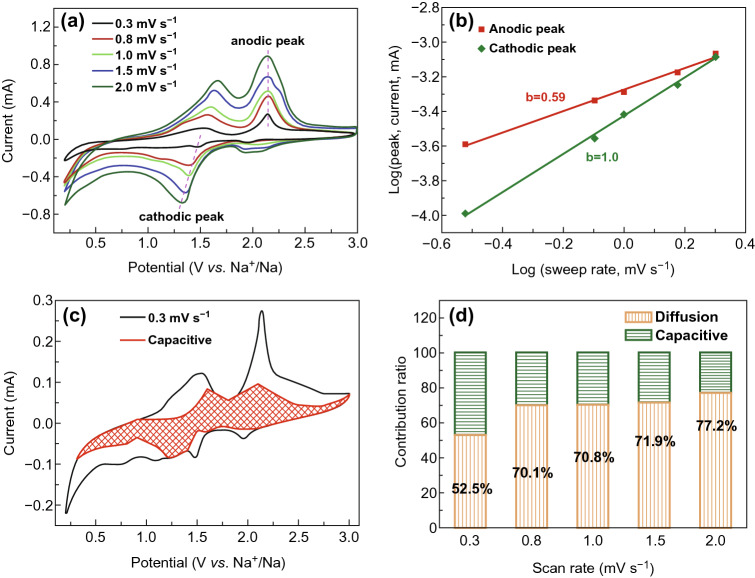
The capacitance effect resulted from VS_4_ and the separation from capacitive and diffusion controlled. **a** CV scan from 0.3 to 2 mV s^−1^, **b** *b* value issued from the relationship between peak current and CV scan rates, **c** current separation arising from capacitive and diffusion controlled, **d** the total capacity contribution ratio of capacitive effect and diffusion controlled at different scan rates

The reaction mechanism of the Faraday energy storage device involves two distinct processes, including diffusion-controlled (battery) and surface-controlled (capacitive) process. To evaluate the contribution of pseudocapacitance, a linear relationship between the current of peaks and scan rates from 0.3 to 2 mV s^−1^ was recorded and is presented in Fig. 7a. It can be seen that with the increase in the scanning speed, the curve shows a similar shape during the discharging and charging processes at each scan rate and shows a small peak shift indicating the relatively low polarization of VS_4_ in the ester-based electrolyte. The linear relationship between the measured peaks current (*i*) and the scan rate (*v*) can be calculated by Eq. 4 [55]:

4 $$i = av^{b}$$

where *a* and *b* are adjustable parameters. The *b* value of 0.5 indicates that the dominant process is diffusion-controlled (battery) process, and the *b* value of 1 can be attributed to surface-controlled (capacitive) process [56, 57]. Figure 7b shows the log(*v*)–log(*i*) profile of the VS_4_ electrode, where the *b* value can be obtained by fitting a slope. For sweep rates ranging from 0.3 to 2 mV s^−1^, the *b* value for anodic (Peak 2) and cathodic peaks (Peak 1) is 0.59 and 1.0, respectively, indicating that it is the mainly effect of diffusion-controlled process. The contributions of these two processes can be investigated based on Eq. 5:

5 $$i\left( v \right) = k_{1} v + k_{2} v^{{{\raise0.7ex\hbox{$1$} \!\mathord{\left/ {\vphantom {1 2}}\right.\kern-0pt} \!\lower0.7ex\hbox{$2$}}}}$$

where $$k_{1} v$$ and $$k_{2} v^{{{\raise0.7ex\hbox{$1$} \!\mathord{\left/ {\vphantom {1 2}}\right.\kern-0pt} \!\lower0.7ex\hbox{$2$}}}}$$ are the current contributions derived for the surface-controlled and diffusion-controlled reactions, respectively. As shown in Fig. 7c, 52.5% of the total capacity at the sweep rate of 0.3 mV s^−1^ was provided by pseudocapacitance. As the scan rate increased from 0.3 to 3 mV s^−1^, the contribution of pseudocapacitors increased from 52.5 to 77.2% (Fig. 7d). Accordingly, the stored capacity of the battery is mainly owed to the capacitance. Since the unique hairball-like structure has a larger specific surface area than nanosphere, it can provide more active sites and short diffusion pathways for the fast Na^+^ diffusion and good electrolyte accessibility for all electroactive surfaces. Thus, it can be concluded that this unique structure contributes to high pseudocapacitance.

## Conclusions

In summary, pure hairball-like VS_4_ materials were successfully synthesized via an environmentally hydrothermal treatment method. Hairball-like VS_4_ as the anodes for SIBs showed the high reversible capacities, excellent electrochemical performance at room temperature as well as at 0 °C. It was observed that the Na^+^ storage mechanism of VS_4_ electrodes is composed by a three-step separation mechanism: First, the transformation of VS_4_–Na_2_S and V is partially reversible during the initial ten cycles; second, the increase in capacities can owe to the increment of the reversible conversion reaction between Na_2_S and S to store Na^+^; and last, stabilization stage can be described by reversible transformation reaction between Na_2_S and S which was the main reaction mechanism of Na–S batteries. The prepared VS_4_ possesses a high Na^+^ storage capacity after 100 cycles which can be attributed to the low *R*_ct_ and Warburg factor, and the high ionic conductivity values, which were the explanations for the decrease in the first few cycles and increase in the subsequent cycles of capacities. It is believed that the present research on the hairball-like VS_4_ can provide essential ideas for the investigations of other chalcogenides in fields of batteries, catalysts, and electronic devices.

## Electronic supplementary material

Below is the link to the electronic supplementary material.

Supplementary material 1 (PDF 825 kb)
