# Supplementary material for: Three-Dimensional Self-assembled Hairball-Like VS4 as High-Capacity Anodes for Sodium-Ion Batteries
Source: Nanomicro Lett. 2020 Jan 25;12:39. doi: 10.1007/s40820-020-0377-7 (PMC7770669; doi:10.1007/s40820-020-0377-7)
Supplement: Supplementary file 1 — Supplementary material 1 (PDF 825 kb) [file 40820_2020_377_MOESM1_ESM.pdf]

Supporting Information for

Three-dimensional Self-assembled Hairball-like VS_4 as High Capacity Anodes for Sodium-ion Batteries

Shuangshuang Ding[1], Bingxin Zhou[1], Changmiao Chen[1], Zhao Huang[2], Pengchao Li[1], Shuangyin Wang[3], Guozhong Cao[4], Ming Zhang[1, *]

[1]Key Laboratory for Micro/Nano Optoelectronic Devices of Ministry of Education, Hunan Provincial Key Laboratory of Low-Dimensional Structural Physics and Devices, School of Physics and Electronics, Hunan University, Changsha 410082, People's Republic of China

[2]College of Electrical Engineering & New Energy, Three Gorges University, Yichang, Hubei 443002, People's Republic of China

[3]State Key Laboratory of Chemo/Biosensing and Chemometrics, Provincial Hunan Key Laboratory for Graphene Materials and Devices, College of Chemistry and Chemical Engineering, Hunan University, Changsha 410082, People's Republic of China

[4]Department of Materials Science & Engineering, University of Washington, Seattle, WA 98195, USA

*Corresponding author. E-mail: zhangming@hnu.edu.cn (Ming Zhang)

Supplementary Figures and Table

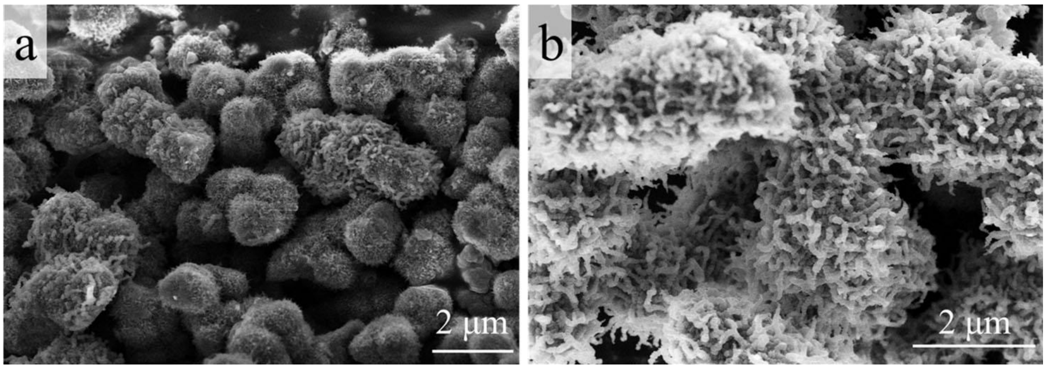

Fig. S1 The SEM images of VS_4 with different heats times. (**a**) 30 h, (**b**) 45 h

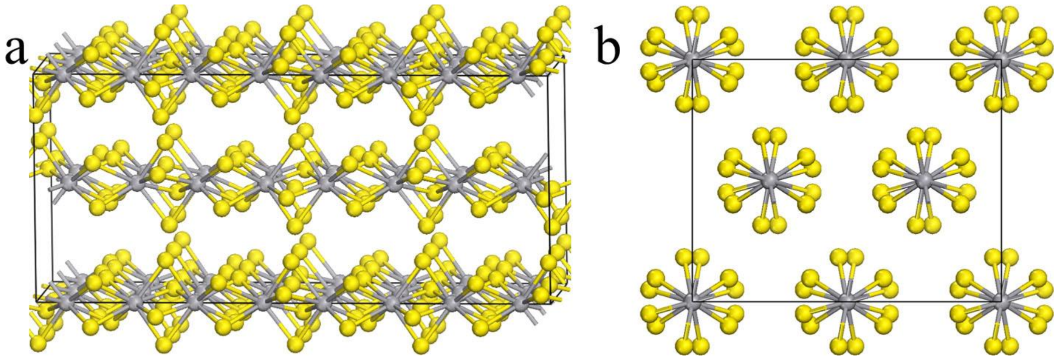

Fig. S2 Schematic geometries of VS_4 nanostructures. The lateral-view and vertical-view of VS_4

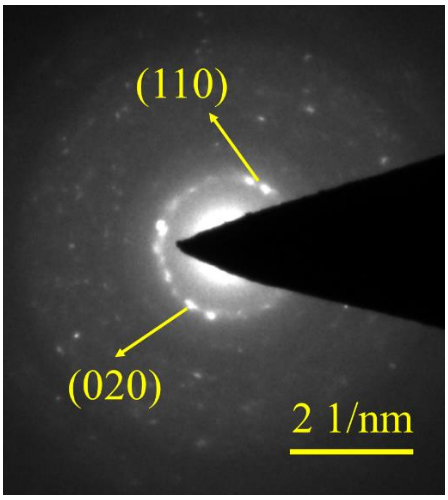

Fig. S3 Selected area electron diffraction (SAED) pattern of hairball-like VS_4

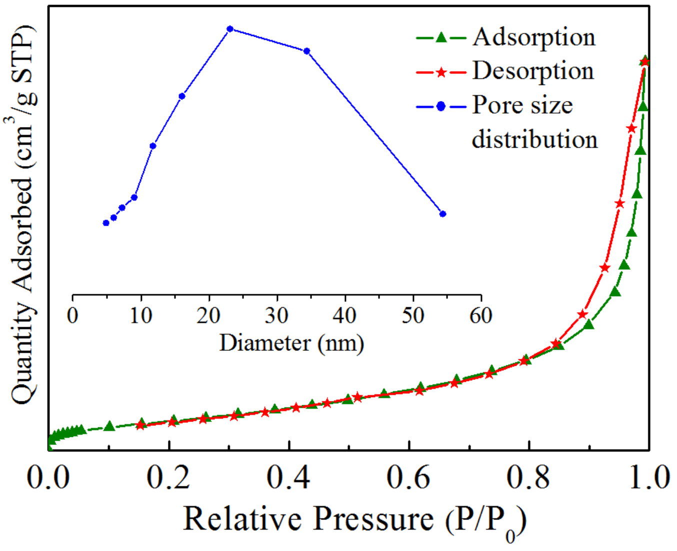

Fig. S4 N_2 adsorption-desorption isotherm and pore size distribution of the hairball-like VS_4

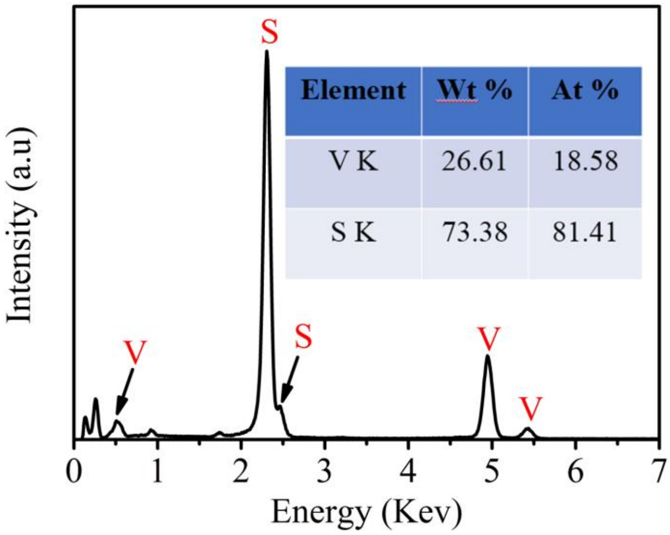

Fig. S5 Energy dispersive spectrometer analysis of hairball-like VS_4

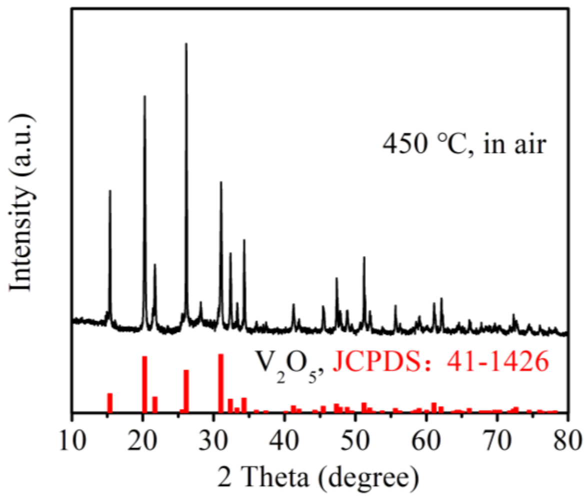

Fig. S6 XRD pattern of the product of the VS_4 heated to 450 °C in air

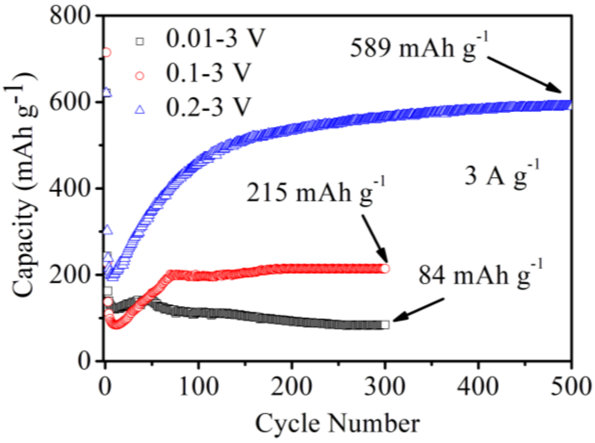

Fig. S7 Cycling properties of VS_4 with different cut-off voltages at a current density of 3 A g^{-1}

During the charging/discharging period, the low cut-off potential provides a relatively high capacity; however, this causes the decomposition of active materials, formation of dendrites, and deterioration of electrochemical performance. Therefore, a suitable cut-off potential can provide extremely long-term stability of batteries. In Fig. S6, although a high initial charging capacity of 714 mAh g^{-1} was observed in the cut-off voltage of 0.01-3.0 V, the capacity rapidly decayed. Furthermore, the cyclical stabilities of batteries in cut-off voltage of 0.1-3 V was not significantly improved. In short, a cut-off voltage of 0.2-3 V was selected for the subsequent study.

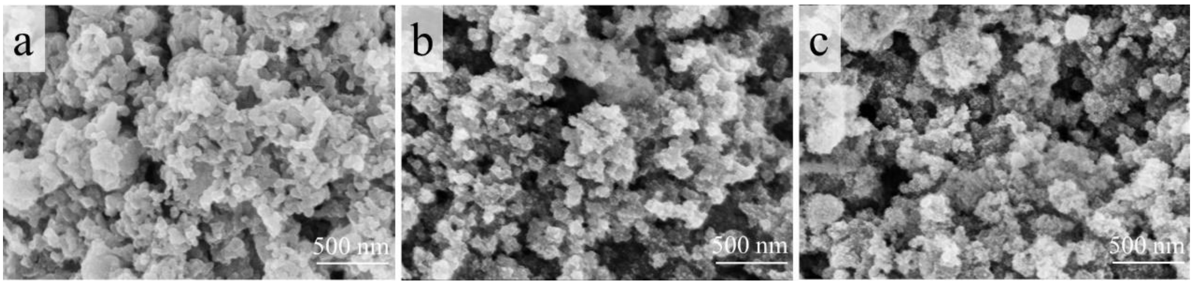

Fig. S8 The SEM images of VS_4 electrode after different cycle numbers. (**a**) 0 cycles,

(**b**) 50 cycles, (**c**) 100 cycles

Table S1 The Na^+ storage performance of the V-based sulfide

Materials	*Voltage range (V vs. Na^+/Na)*	*Capacity (mAh/g) / current density (A/g) / cycles*	*Rate capacity (mAh/g)/ current density (A/g)*	*Refs.*
VS_2 microrods	0.5-3	350/0.2/200	140/0.1; 105/0.2; 77/0.5; 60/1; 50/2; 35/5	[S1]
VS_2 nanosheets	0.3-3	386/0.1	\	[S2]
VS_4/GS nanocomposites	0.3-3	349/0.1/100	\	[S3]
VS_4-rGO composite	0.01-3	402/0.5/300	605/0.1; 547/0.2; 507/0.4; 469/0.6; 460/0.8; 446/1	[S4]
VS_2 nanoarchitectures	0.5-3	403/0.2/200	258/0.1; 231/0.2; 193/0.5; 172/1; 141/2	[S5]
VS_4 microspheres	0.5-3	412/0.2/230	408/0.2; 370/0.5; 345/1; 293/2; 201/5	[S6]
VS_2-SNSs	0.4-2.2	204/5/600	252/0.1; 203/5; 180/10	[S7]
c-VS_2@VOOH	0.5-3	330/0.2/150	424/0.1; 404/0.2; 356/0.5; 224/1; 140/2; 113/5	[S8]
VS_4-G nanocomposite	0.01-3	463/0.1/100	482/0.2; 408/0.6; 345/1.2; 270/2.4	[S9]
VS_4/rGO	0.01-2.2	241/0.1/50	342/0.1; 267/0.2; 220/0.5; 192/0.8	[S10]
flower-like VS_2	0.3-3	600/0.1/50	352/10; 277/20	[S11]
VS_4 microspheres	0.01-3	302/0.2/120	686/0.05; 496/0.2; 453/1	[S12]
VS_2 nanosheets	0.3-3	565/0.2/1000	750/0.2; 651/0.5; 598/1; 567/2; 533/4	[S13]
VS_4 nanoarchitectures	0.5-3	225/0.5/200	265/0.2; 229/0.5; 203/1; 168/2; 122/5	[S14]

Supplementary References

[S1]W. Li, J. Huang, L. Feng, L. Cao, Y. Liu, L. Pan, Nano-grain dependent 3D hierarchical VS_2 microrods with enhanced intercalation kinetic for sodium storage properties. J. Power Sources **398**, 91-98 (2018). https://doi.org/10.1016/j.jpowsour.2018.07.036

[S2]D. Wang, Y. Zhao, R. Lian, D. Yang, D. Zhang, X. Meng, Y. Liu, Y. Wei, G. Chen, Atomic insight into the structural transformation and anionic/cationic redox reactions of VS_2 nanosheets in sodium-ion batteries. J. Mater. Chem. A **6**(33), 15985-15992 (2018). https://doi.org/10.1039/C8TA05396K

[S3]Q. Pang, Y. Zhao, Y. Yu, X. Bian, X. Wang, Y. Wei, Y. Gao, G. Chen, Ultrafine VS_4 Nanoparticles Anchored on Graphene Sheets as a High-Rate and Stable

Electrode Material for Sodium Ion Batteries. Chemsuschem **11**(4), 735-742 (2018). https://doi.org/10.1002/cssc.201702031

[S4]S. Wang, F. Gong, S. Yang, J. Liao, M. Wu, Z. Xu, C. Chen, X. Yang, F. Zhao, B. Wang, Y. Wang, X. Sun, Graphene Oxide-Template Controlled Cuboid-Shaped High-Capacity VS_4 Nanoparticles as Anode for Sodium-Ion Batteries. Adv. Funct. Mater. **28**(34), 1801806 (2018). https://doi.org/10.1002/adfm.201801806

[S5]W. Li, J. Huang, L. Feng, L. Cao, Y. Liu, L. Pan, VS_2 nanoarchitectures assembled by single-crystal nanosheets for enhanced sodium storage properties. Electrochim. Acta **286**, 131-138 (2018). https://doi.org/10.1016/j.electacta.2018.08.049

[S6]W. Li, J. Huang, L. Feng, L. Cao, S. He, 3D self-assembled VS_4 microspheres with high pseudocapacitance as highly efficient anodes for Na-ion batteries. Nanoscale **10**(46), 21671-21680 (2018). https://xs.scihub.ltd/10.1039/C8NR06458J

[S7]R. Sun, Q. Wei, J. Sheng, C. Shi, Q. An, S. Liu, L. Mai, Novel layer-by-layer stacked VS_2 nanosheets with intercalation pseudocapacitance for high-rate sodium ion charge storage. Nano Energy **35**, 396-404 (2017). https://doi.org/10.1016/j.nanoen.2017.03.036

[S8]W. Li, J. Huang, L. Feng, L. Cao, Y. Feng, H. Wang, J. Li, C. Yao, Facile in situ synthesis of crystalline VOOH-coated VS_2 microflowers with superior sodium storage performance. J. Mater. Chem. A **5**(38), 20217-20227 (2017). https://doi.org/10.1039/C7TA05205G

[S9]S. Li, W. He, P. Deng, J. Cui, B. Qu, Ultra-long cycle life of sodium-ion batteries in VS_4-G nanocomposite structure. Mater. Lett. **205**, 52-55 (2017). https://doi.org/10.1016/j.matlet.2017.06.058

[S10] R. Sun, Q. Wei, Q. Li, W. Luo, Q. An, J. Sheng, D. Wang, W. Chen, L. Mai, Vanadium Sulfide on Reduced Graphene Oxide Layer as a Promising Anode for Sodium Ion Battery. ACS Appl. Mater. Interfaces **7**(37), 20902-20908 (2015). https://doi.org/10.1021/acsami.5b06385

[S11] D. Yu, Q. Pang, Y. Gao, Y. Wei, C. Wang, G. Chen, F. Du, Hierarchical flower-like VS_2 nanosheets–A high rate-capacity and stable anode material for sodium-ion battery. Energy Storage Mater. **11**, 1-7 (2018). https://doi.org/10.1016/j.ensm.2017.09.002

[S12] W. Li, J. Huang, L. Feng, L. Cao, Y. Liu, L. Pan, VS_4 microspheres winded by (110)-oriented nanotubes with high rate capacities as sodium-ion battery anode. Mater. Lett. **230**, 105-108 (2018). https://doi.org/10.1016/j.matlet.2018.07.101

[S13] J. Wang, N. Luo, J. Wu, S. Huang, L. Yu, M. Wei, Hierarchical spheres constructed by ultrathin VS_2 nanosheets for sodium-ion batteries. J. Mater. Chem. A **7**(8), 3691-3696 (2019). https://doi.org/10.1039/C8TA11950C

[S14] W. Li, J. Huang, L. Cao, L. Feng, C. Yao, Controlled construction of 3D self-assembled VS_4 nanoarchitectures as high-performance anodes for sodium-ion batteries. Electrochim. Acta **274**, 334-342 (2018). https://doi.org/10.1016/j.electacta.2018.04.106
